# Host specificity and zoonotic *Enterocytozoon bieneusi* genotypes in wild rodents from the Inner Mongolian Autonomous Region and Liaoning Province of China

**DOI:** 10.3389/fcimb.2024.1409685

**Published:** 2024-06-18

**Authors:** Wei Zhao, Lijie Sun, Li Liu, Aiying Jiang, Qiang Xiao, Feng Tan

**Affiliations:** ^1^ School of Basic Medical Sciences, Wenzhou Medical University, Wenzhou, Zhejiang, China; ^2^ Department of Clinical Laboratory, The Fifth Affiliated Hospital, Sun Yat-sen University, Zhuhai, China; ^3^ Department of Public Health and Laboratory Medicine, Yiyang Medical College, Yiyang, China

**Keywords:** *Enterocytozoon bieneusi*, rodent, genotype, ITS, zoonotic

## Abstract

**Introduction:**

Wild rodents can serve as reservoirs or carriers of *E. bieneusi*, thereby enabling parasite transmission to domestic animals and humans. This study aimed to investigate the prevalence of *E. bieneusi* in wild rodents from the Inner Mongolian Autonomous Region and Liaoning Province of China. Moreover, to evaluate the potential for zoonotic transmission at the genotype level, a genetic analysis of the isolates was performed.

**Methods:**

A total of 486 wild rodents were captured from two provinces in China. Polymerase chain reaction (PCR) was performed to amplify the vertebrate *cytochrome b* (*cytb*) gene in the fecal DNA of the rodents to detect their species. The genotype of *E. bieneusi* was determined *via* PCR amplification of the internal transcribed spacer (ITS) region of rDNA. The examination of genetic characteristics and zoonotic potential requires the application of similarity and phylogenetic analysis.

**Results:**

The infection rates of *E. bieneusi* in the four identified rodent species were 5.2% for *Apodemus agrarius* (n = 89), 4.5% for *Cricetulus barabensis* (n = 96), 11.3% for *Mus musculus* (n = 106), and 38.5% for *Rattus norvegicus* (n = 195). Infection was detected at an average rate of 17.4% among 486 rodents. Of the 11 identified genotypes, nine were known: SHR1 (detected in 32 samples), D (30 samples), EbpA (9 samples), PigEbITS7 (8 samples), HNR-IV (6 samples), Type IV (5 samples), HNR-VII (2 samples), HNH7 (1 sample), and HNPL-V (1 sample). Two novel genotypes were also discovered, NMR-I and NMR-II, each comprising one sample. The genotypes were classified into group 1 and group 13 *via* phylogenetic analysis.

**Discussion:**

Based on the initial report, *E. bieneusi* is highly prevalent and genetically diverse in wild rodents residing in the respective province and region. This indicates that these animals are crucial for the dissemination of *E. bieneusi*. Zoonotic *E. bieneusi*-carrying animals present a significant hazard to local inhabitants. Therefore, it is necessary to increase awareness regarding the dangers presented by these rodents and reduce their population to prevent environmental contamination.

## Introduction

*Enterocytozoon bieneusi* is an obligate intracellular pathogen mainly observed in immunocompromised patients, especially those with HIV, who suffer from chronic or persistent diarrhea, colic, vomiting, and/or anorexia (Han et al., 2021). Recently, there has been a growing number of reports indicating its presence in asymptomatic healthy persons (Han et al., 2021). However, their route of transmission is still not fully understood. The widespread distribution of infectious spores in nature and their ability to infect nearly all animal kingdoms have led to the hypothesis of zoonotic transmission (Ruan et al., 2021). Based on this presumption, the fecal-oral route is the main mode of transmission, either through infected animal-to-human contact or through the ingestion of contaminated food and water (Li and Xiao, 2020a). Outbreaks of this pathogen that are predominantly transmitted *via* food and water have been documented (Decraene et al., 2012; Bourli et al., 2023). *E. bieneusi* has a wide influence, being classified as a Category B Priority Pathogen by the National Institute of Allergy and Infectious Diseases and designated by the US Environmental Protection Agency as a potential water-borne contaminant (EPA, 1998; Didier and Weiss, 2006). As there are currently no targeted treatments or vaccines available to control microsporidiosis, it is crucial to comprehend the origins and methods of transmission of this disease.

*E. bieneusi* has been successfully identified by molecular diagnostic techniques employing PCR, thereby contributing significantly to the field of epidemiology. Currently, the most commonly employed technique is ribosomal internal transcriber spacer (ITS) nucleotide sequence amplification and sequencing analysis (Santín and Fayer, 2009). More than 850 unique genotypes of *E. bieneusi* have been identified through this method, and comprehensive data on the distribution of genotypes among animal and human hosts have been obtained, with at least 126 of them being found exclusively in humans (Koehler et al., 2022). Moreover, the detection of 58 genotypes in both humans and animals provides further evidence for the occurrence of zoonotic transmission (Koehler et al., 2022). Phylogenetic analysis is also crucial for evaluating the zoonotic potential of genotypes. Based on the current study, the identified genotypes can be categorized into 15 distinct clusters referred to as groups 1 to 15 (Zhao et al., 2020; Jiang et al., 2024). Importantly, groups 1 and 2 were identified as the most significant, accounting for 90% of the genotypes. As these two groups constitute the majority of zoonotic genotypes, they are designated zoonotic groups (Li et al., 2019a). Therefore, genotypes associated with these groups show a greater possibility of potential comoronotic transmission. Conversely, the genotypes comprising the 13 remaining groups revealed a substantially higher level of host specificity, thereby limiting the potential for zoonotic transmission (Li et al., 2019a). Currently, the precise role of each host in the transmission of the disease remains enigmatic. Thus, to effectively limit the widespread prevalence of *E. bieneusi*, it is crucial to study and examine a wide range of hosts, especially those that are close to humans.

Growing evidence indicates that wild animals are essential for the transmission and hosting of *E. bieneusi* genotypes, which can adapt to hosts and transmit diseases to humans (Koehler et al., 2022). Rodents have been identified as potential carriers or reservoirs of *E. bieneusi*, and they disseminate the parasite among humans and other animals in both rural and urban areas (Taghipour et al., 2022). The typing data indicate that these rodents harbor more than 100 genotypes of *E. bieneusi*, with significant overlap in genotypes in humans (Zhao et al., 2023). This overlap emphasizes the relevance of rodents as a crucial factor in *E. bieneusi* transmission to humans and the necessity of incorporating them into efforts to eradicate this pathogen.

China is a biodiverse hub for rodents, with a total of 235 species from 12 different families. Significantly, this pathogen is distributed in 16 provinces and has been detected in 22 of these rodent species (Ni et al., 2021; Tuo et al., 2023; Zhao et al., 2023; Jiang et al., 2024). This comprises a wide range of rodent species, including domesticated and wild rodents, experimental subjects, and even household pets (Zhao et al., 2023). Despite these developments, the understanding of the transmission of *E. bieneusi* infections among different host species is still incomplete. Importantly, epidemiological data on this topic are particularly limited in the Inner Mongolian Autonomous Region and Liaoning Province of China. Therefore, the current study evaluated the zoonotic potential of *E. bieneusi* isolates at the genotype level by observing their prevalence in rodents from the respective regions.

## Materials and methods

### Ethical consideration

The protocols of the present study were approved by the Research Ethics Committee of Wenzhou Medical University after a rigorous review procedure with approval number SCILLSC-2021–01.

### Sample collection

A total of 486 wild rodents were collected between November 2023 and February 2024 from two distinct regions: Jianping County of Liaoning Province and Harqin Banner of Inner Mongolia, China, which contributed 229 and 257 rodents, respectively. The rodents were captured using cage traps that were loaded with a combination of peanut and sunflower seeds. To establish transects, approximately 50 cage traps were positioned in a straight line along each designated capture location, with a consistent spacing of 5 m between each trap. The transects were positioned precisely at 4:00 PM and reconvened the next morning at 8:00 AM. Each captured rodent was euthanized *via* CO_2_ asphyxiation and immediately transported to the laboratory in containers comprising ice, ensuring its safety for 48 h. A fecal sample (0.5 g) was collected from the rectum of each rodent.

### DNA extraction

The collected samples (0.2 g) were processed for DNA extraction, while the remainder of the sample was kept as a backup and stored at -80°C. Genomic DNA was isolated from each processed sample *via* the QIAamp DNA Mini Stool Kit (Qiagen, Germany). The lysis temperature was increased to 95°C during extraction, and all other procedures were carried out under strict guidelines provided by the manufacturer. The DNA was then reconstituted in 200 µL of AE elution buffer, which was included in the reagent, and stored at -20 °C before PCR analysis.

### Identification of rodent species

The vertebrate *cytochrome b* (*cytb*) gene (421 bp) was amplified from the fecal DNA using PCR to identify the rodent species. Based on the methodology described by Verma and Singh (2003), the primer sequences utilized were 5’-TACCATGAGGACAAATATCATTCTG-3’ and 5’-CCTCCTAGTTTGTTAGGGATTGATCG-3’. The PCR parameters were as follows: 35 cycles of denaturation (94 °C for 30 s), annealing (51 °C for 30 s), and extension (72 °C for 30 s). Prior to this, an initial denaturation step was performed at 94°C for 5 minutes, followed by a final extension step at 72 °C for an additional 5 minutes.

### Genotyping of *E. bieneusi*

The genotype of E. bieneusi was determined through the amplification of the ITS region using nested PCR, employing primers and cycle parameters established by Buckholt et al. Specifically, the external PCR primers used were EBITS3 (5’-GGT CAT AGG GAT GAA GAG-3’) and EBITS4 (5’-TTC GAG TTC TTT CGC GCT C-3’), while the internal PCR primers used were EBITS1 (5’-GCT CTG AAT ATCTAT GGC T-3’) and EBITS2.4 (5’-ATC GCC GAC GGATCC AAG TG-3’). The cycling parameters involved two distinct sets: the first set consisted of 35 cycles, with denaturation at 94°C for 30 seconds, annealing at 57°C for 30 seconds, and extension at 72°C for 40 seconds. The second set comprised 30 cycles of denaturation at 94°C for 30 seconds, annealing at 55°C for 30 seconds, and extension at 72°C for 40 seconds. Both sets concluded with a final extension step at 72°C for 10 minutes. TaKaRa Taq DNA Polymerase was used in combination with genotype BEB6 DNA from deer as a positive control and 2 μL of distilled water as a negative control. The PCR results were further examined using 1.5% agarose gel electrophoresis and then visualized *via* DNAGREEN staining (Tiandz, Inc., China).

### DNA sequencing and analysis

The PCR products that yielded positive results for *E. bieneusi* were sequenced *via* bidirectional sequencing (Sangon Biotech Co., Ltd., China). The Basic Local Alignment Search Tool (BLAST) and ClustalX 1.83 software were used for genotyping the *E. bieneusi* isolates. This process involved comparing the identified nucleotide sequences with published GenBank sequences. The genotypes were designated using the standard nomenclature system based on 243 bp of the ITS region of *E. bieneusi* (Santín and Fayer, 2009). Specifically, if the nucleotide sequences were identical to known genotypes, the first published name was assigned. Conversely, if the nucleotide sequences differed from those previously published and were verified as novel sequences through sequencing of two additional separate PCR products derived from the same preparations, they would represent distinct, novel genotypes.

### Phylogenetic analysis

A neighboring-joining phylogenetic tree was generated *via* the Kimura-2-parameter model using Mega X software. Moreover, to validate the gene groups and examine the associations between their genotypes, 1,000 replicates were performed.

### Statistical analyses

To determine the disparities in *E. bieneusi* prevalence across rodent species and regions, the chi-square test was applied to each of the two variables. P values ≤ 0.05 were considered to indicate statistical significance.

### Nucleotide sequence accession numbers

The GenBank database accession numbers of the detected nucleotide sequences are PP550151 to PP550161.

## Results

### Rodent species identification

In this study, four different rodent species, namely, *Apodemus agrarius* (n = 96), *Cricetulus barabensis* (n = 89), *Mus musculus* (n = 106), and *Rattus norvegicus* (n = 195), were identified by sequencing analysis of the cytb gene after PCR. No additional information were collected on these wild rodents (**Table 1**).

**Table 1:** Prevalence and distribution of *E. bieneusi* genotype in the investigated rodents from the inner mongolian autonomous region and liaoning province of China.

Rodent species	Inner Mongolia (Harqin Banner)		Liaoning (Jianping)		Total	
	Positive/examined (%)	*E. bieneusi* genotype (n)	Positive/examined (%)	*E. bieneusi* genotype (n)	Positive/examined (%)	*E. bieneusi* genotype (n)
*Apodemus agrarius*	2/36 (5.6)	D (1), SHR1 (1)	3/60 (5.0)	SHR1 (3)	5/96 (5.2)	SHR1 (4), D (1)
*Cricetulus barabensis*	3/62 (4.8)	D (3)	1/27 (3.7)	SHR1 (1)	4/89 (4.5)	D (3), SHR1 (1)
*Mus musculus*	8/28 (28.6)	D (3), PigEbITS7 (3), HNPL-V (1), Type IV (1)	4/78 (5.1)	Type IV (2), HNR-VII (2)	12/106 (11.3)	D (3), PigEbITS7 (3), Type IV (3), HNR-VII (2), HNPL-V (1)
*Rattus norvegicus*	49/103 (47.6)	SHR1 (22), D (8), EbpA (6), HNR-IV (6), PigEbITS7 (5), NMR-I (1), NMR-II (1)	26/92 (28.3)	D (15), SHR1 (5), EbpA (3), Type IV (2), HNH7 (1)	75/195 (38.5)	SHR1 (27), D (23), EbpA (9), HNR-IV (6), PigEbITS7 (5), Type IV (2), HNH7 (1), NMR-I (1), NMR-II (1)
Total	62/229 (27.1)	SHR1 (23), D (15), PigEbITS7 (8), EbpA (6), HNR-IV (6), Type IV (1), HNPL-V (1), NMR-I (1), NMR-II (1)	34/257 (13.2)	D (15), SHR1 (9), Type IV (4), EbpA (3), HNR-VII (2), HNH7 (1)	96/486 (19.6)	SHR1 (32), D (30), EbpA (9), PigEbITS7 (8), HNR-IV (6), Type IV (5), HNR-VII (2), HNH7 (1), HNPL-V (1), NMR-I (1), NMR-II (1)

### Prevalence of *E. bieneusi*

Among all 486 tested samples, *E. bieneusi* was detected in 19.6% (96 samples). The infection rates varied among the different species. For example, *A. agrarius* had an infection rate of 5.2% (5/96), *C. barabensis* had a rate of 4.5% (4/89), *M. musculus* had a rate of 11.3% (12/106), and *R. norvegicus* had a particularly high rate of 38.5% (75/195) (**Table 1**). These results demonstrated significant differences in the rates of infection in various rodent species (χ^2^ = 73.7; df = 3; *P <*0.001). Moreover, a comparison between the two regions showed that animals from Inner Mongolia had a much greater infection rate (27.1%, 62/229) than those from Liaoning (13.2%, 34/257) (χ^2^ = 14.6; df = 1; *P <*0.001). This suggests that there is a geographical difference in the prevalence of *E. bieneusi.*

### Characterization and distribution of the genotypes of *E. bieneusi*

A total of 11 genotypes were identified after sequencing all 96 samples that tested positive for *E. bieneusi*. Of these, there were nine known genotypes: SHR1 (found in 32 samples), D (30 samples), EbpA (9 samples), PigEbITS7 (8 samples), HNR-IV (6 samples), Type IV (5 samples), HNR-VII (2 samples), HNH7 (1 sample), and HNPL-V (1 sample). Furthermore, this study identified two new genotypes, NMR-I and NMR-II, in each single sample. When comparing the NMR-I (PP550160) genotype to the SHR1 genotype, two single-base differences were observed. Specifically, there was an A to G transition at the 159th nucleotide site and a T to C transition at the 209th nucleotide site. However, there was minor variation between NMR-II (PP550160) and genotype D, with the addition of a G base at the 14th nucleotide site.

There was some variation in the distribution of *E. bieneusi* genotypes among all rodent species. Genotype D was found in all four wild rodent species, whereas genotype SHR1 was detected in three rodent species, except *C. barabensis*. Conversely, genotypes PigEbITS7 and Type IV were limited to two rodent species, *M. musculus* and *R. norvegicus*. The HNR-VII and HNPL-V genotypes were exclusively found in *M. musculus*, while the EbpA, HNR-IV, HNH7, NMR-I and NMR-II genotypes were uniquely found in *R. norvegicus* (**Table 1**).

### Phylogenetic analysis

**Figure 1:**
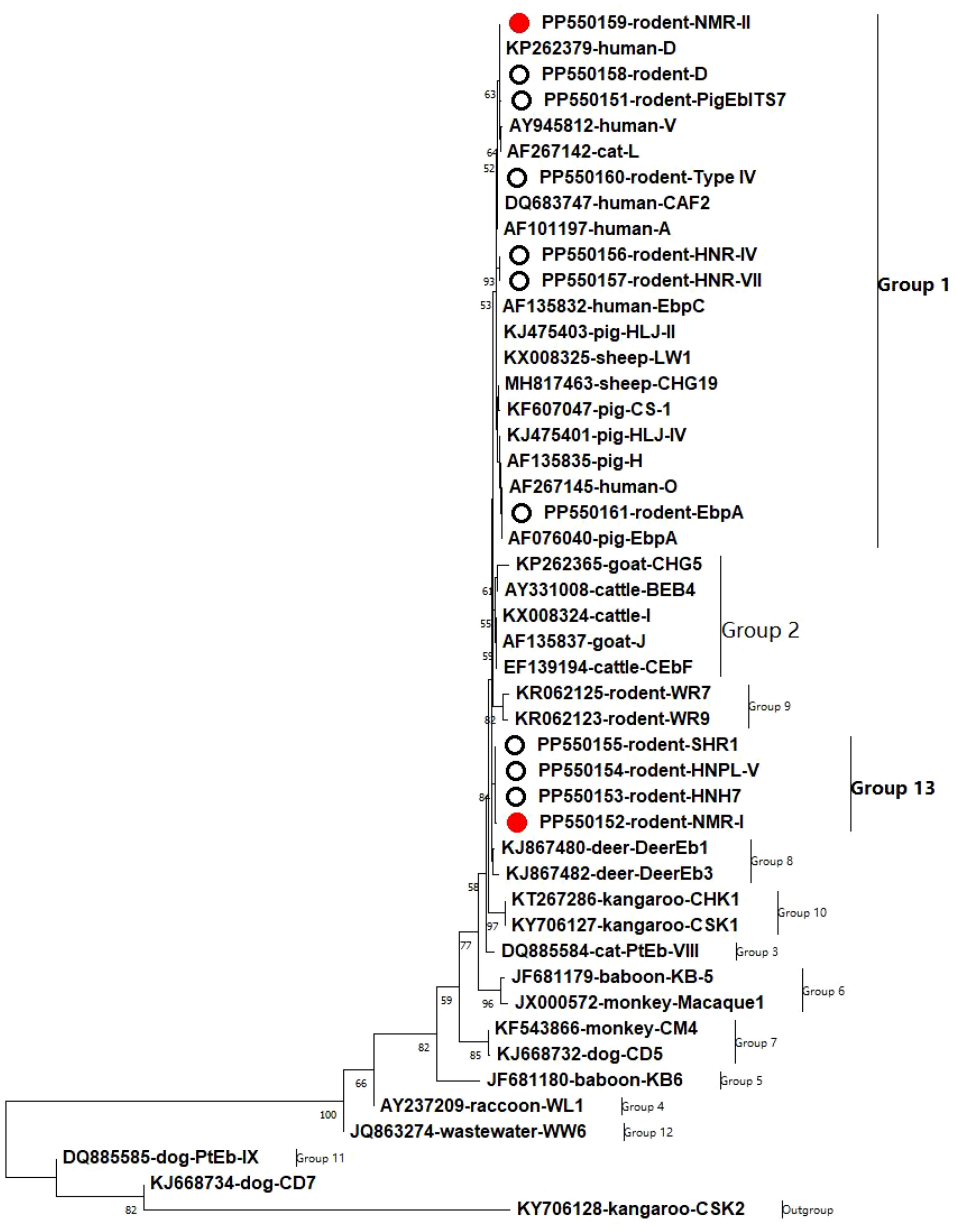
A phylogenetic tree was constructed to represent the genetic relationships among various *E. bieneusi* genotypes, based on their ITS sequences. This tree was generated using the neighboring-joining method, which relies on the Kimura-2-parameter model. To assess the reliability of the tree, bootstrap values were derived from 1,000 replicates. In this tree, genotypes are distinguished by hollow circles and red hollow circles, indicating known and novel sequences identified in this study, respectively.

The identified genotypes in the ITS region of *E. bieneusi* were classified into two different clusters based on phylogenetic analysis: Group 1, which consisted of seven genotypes, and Group 13, which contained four genotypes (**Figure 1**).

## Discussion

This study revealed that the average infection rate of *E. bieneusi* among rodents in the Inner Mongolian Autonomous Region and Liaoning Province of China is 19.6%. This result exceeds the rates recorded in several countries, including Peru (14.9%), the United States (18.0%), the Czech Republic and Germany border (10.7%), China (13.9%), Japan (13.0%), and Slovakia (1.1%). However, the infection rate observed in Poland is higher at 38.9% (Taghipour et al., 2022; Tuo et al., 2023; Zhao et al., 2023). It is crucial to acknowledge that, except for China and the United States, each of these countries has only been the focus of a single study. This constraint significantly undermines the reliability of cross-national comparisons. There is considerable variation in infection rates among distinct regions in China. The findings of this study indicate that Inner Mongolia has a greater infection rate than Liaoning. Conversely, it is lower than the rates reported in Chongqing, Anhui, Henan, and Shanghai but higher than those found in Xinjiang, Hainan, Heilongjiang, and Zhejiang (Ni et al., 2021; Tuo et al., 2023; Zhao et al., 2023; Jiang et al., 2024). Infection rates are significantly influenced not only by geographical differences but also by the species of rodents. The results of this study suggested that *R. norvegicus* had the highest infection rate of 38.5%, whereas *C. barabensis* had the lowest infection rate of 4.5%. Furthermore, previous studies have shown that infection rates vary among species, ranging from Chinchillas (3.6%) to guinea pigs (87.5%) (Taghipour et al., 2022; Tuo et al., 2023; Zhao et al., 2023). These results indicate that rodents, especially wild rodents, are commonly infected with *E. bieneusi* and have a significant impact on the persistence of high levels of endemicity (Zhao et al., 2023). Considering the importance of these findings, it is crucial to carry out more studies on rodent-borne *E. bieneusi* and their potential implications for public health.

The SHR1 genotype, which was present in 33.3% (32 out of 96) of the samples, was the most prevalent among the 11 identified *E. bieneusi* genotypes. In addition to *M. musculus*, this genotype has been identified in all sampled animal populations, demonstrating its wide distribution. It was initially detected in experimental rodents and rabbits in China and has emerged as the prevailing genotype, having been identified in civets covering 7 provinces in China as well as pet snakes in Beijing (Li et al., 2020b, 2020c; Lin et al., 2021; Zhao et al., 2021). Furthermore, it has also been detected in humans from Hainan Province, China (Zhang et al., 2022). These findings suggest that genotype SHR1 has a wide host range and the potential to infect humans. However, considering its discovery only in China, it is still unclear whether it shows any geographical specificity. Further study is expected to provide more clarity on the actual range of hosts for this genotype.

In the present study, genotype D was found to be the second most prevalent genotype after SHR1, at 31.3% (30/96). Multiple studies have consistently demonstrated a heightened occurrence of this genotype among humans, particularly among immunocompromised individuals and those with gastrointestinal disorders (Ruan et al., 2021; Zhao et al., 2022). Furthermore, genotype D has been documented in a diverse range of 68 host species across 38 countries, indicating its widespread distribution (Koehler et al., 2022). Its ability to be detected in environmental samples, including water and vegetables, underscores its extensive ecological reach (Salamandane et al., 2023). The adaptability and resilience of the genotype across diverse environments and hosts underscore its potential as a significant threat to public health. The prevalent occurrence of genotype D among wild rodents, as observed in our study, provides additional evidence of the crucial role played by these animals in the dissemination of *E. bieneusi*.

Type IV, PigEbITS7, EbpA, and PigEbITS7 are zoonotic genotypes and have been frequently observed in humans from Nigeria, Thailand, China, Bangladesh, Egypt, Mozambique, and Congo (Li et al., 2019a; Koehler et al., 2022). These genotypes have a wide range of hosts, including nonhuman primates, domesticated animals, and avian species (Ruan et al., 2021). In particular, these genotypes have been detected in potable source water from Shanghai and in raw wastewater along with vegetable and fruit surfaces from Henan, China (Ma et al., 2016; Ye et al., 2017; Li et al., 2019b). The current study revealed these three genotypes in 22.9% (22/96) of the rodents surveyed, suggesting a substantial possibility of transmission between infected rodents and both humans and other animals.

Genotype HNH7, which was previously found in humans in Hainan Province, China, was not detected in any other animal until this recent discovery (Zhang et al., 2022). However, this study revealed the presence of this genotype in *R. norvegicus* in Liaoning, indicating its ability to infect both humans and animals. In contrast, genotypes HNR-IV, HNR-VII, and HNPL-V have not been detected in humans. However, they have been found in other animal hosts, especially rodents and civets (Zhao et al., 2021, 2023). Moreover, the genotypes NMGH1 in horses, MJ in sheep (MK348513), CPB19 (OQ534110) in giant pandas and PL2 in civets have also been classified as HNR-IV (Li et al., 2020d; Lin et al., 2021). HNR-VII has been detected in Lesser rice field rats and Asiatic brushtailed porcupines from Hainan, China (Zhao et al., 2020, 2023). HNPL-V has been detected in civets in Hainan Province, China (Zhao et al., 2021). In this study, these genotypes were observed in *R. norvegicus* and *Mus musculus*, which shows that these small rodents play a crucial role in the transmission of *E. bieneusi* among wildlife, the environment, and farmed animals.

In this study, two new genotypes were detected, NMR-II, which is classified in group 1 and is the most common and intricate group comprising ≥ 600 genotypes (Koehler et al., 2022). Specifically, group 1 genotypes have been found in various hosts, including humans, and present a significant risk of transmission across other species and zoonotic transmission (Li et al., 2019a). As a result, there is a prediction that genotype NMR-II may have a wider range of hosts and could potentially infect people. However, additional research is needed to evaluate this prediction.

## Conclusions

This study revealed a concerning rate of *E. bieneusi* infection in four different species of wild rodents found in the Inner Mongolian Autonomous Region and Liaoning Province of China. The presence of the human pathogenic genotype HNH7, along with other zoonotic genotypes of *E. bieneusi*, such as genotype D, PigEbITS7, Type IV, and EbpA, as well as the potential zoonotic SHR1 and host-adaptive HNR-IV, HNR-VII, and HNPL-V genotypes, suggests that these rodents may play a crucial role in the epidemiology and transmission of *E. bieneusi* in the region. Therefore, it is essential to take measures to control rodent infestations and improve hygiene and sanitation practices to prevent the transmission of *E. bieneusi* and other zoonotic diseases.

## Data availability statement

The datasets presented in this study can be found in online repositories. The names of the repository/repositories and accession number(s) can be found in the article/supplementary material.

## Ethics statement

The protocols of the present study were approved by the Research Ethics Committee of Wenzhou Medical University after a rigorous review procedure with approval number SCILLSC-2021-01. The study was conducted in accordance with the local legislation and institutional requirements.

## Author contributions

WZ: Conceptualization, Data curation, Formal analysis, Investigation, Methodology, Writing – original draft, Writing – review & editing. LS: Formal analysis, Investigation, Methodology, Writing – original draft, Writing – review & editing. LL: Formal analysis, Resources, Writing – original draft, Writing – review & editing. AJ: Investigation, Methodology, Writing – original draft, Writing – review & editing. QX: Formal analysis, Funding acquisition, Supervision, Writing – original draft, Writing – review & editing. FT: Conceptualization, Data curation, Supervision, Writing – original draft, Writing – review & editing.
